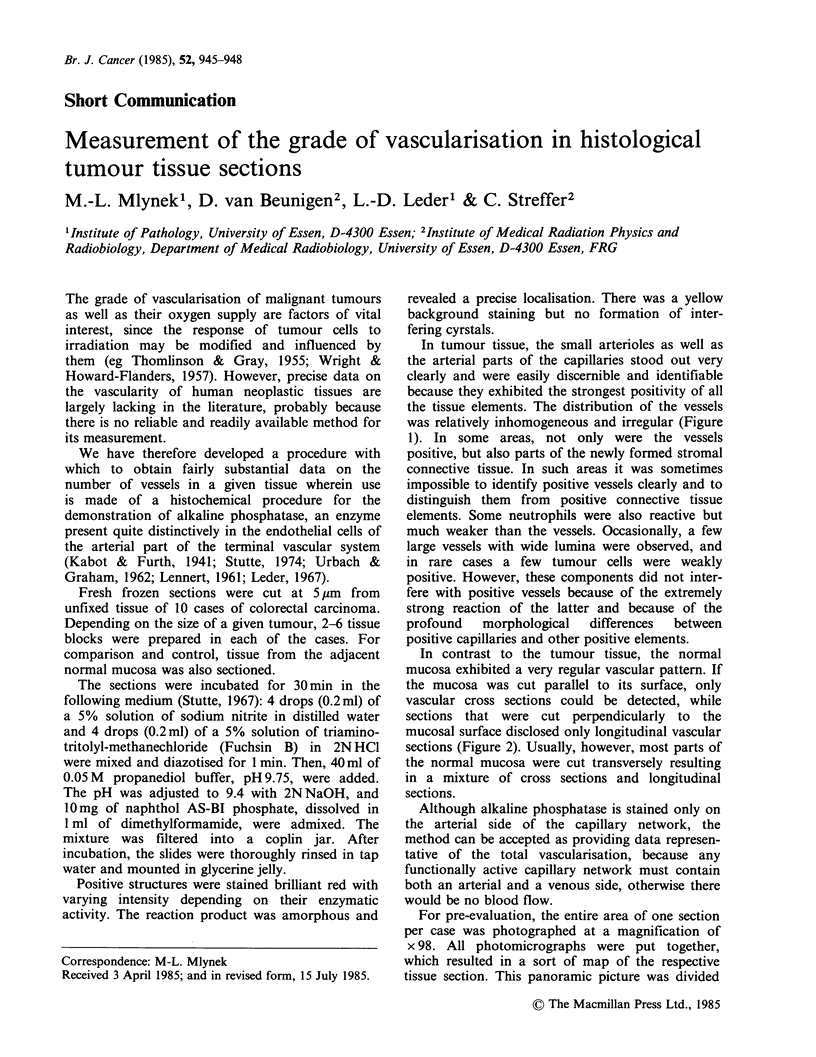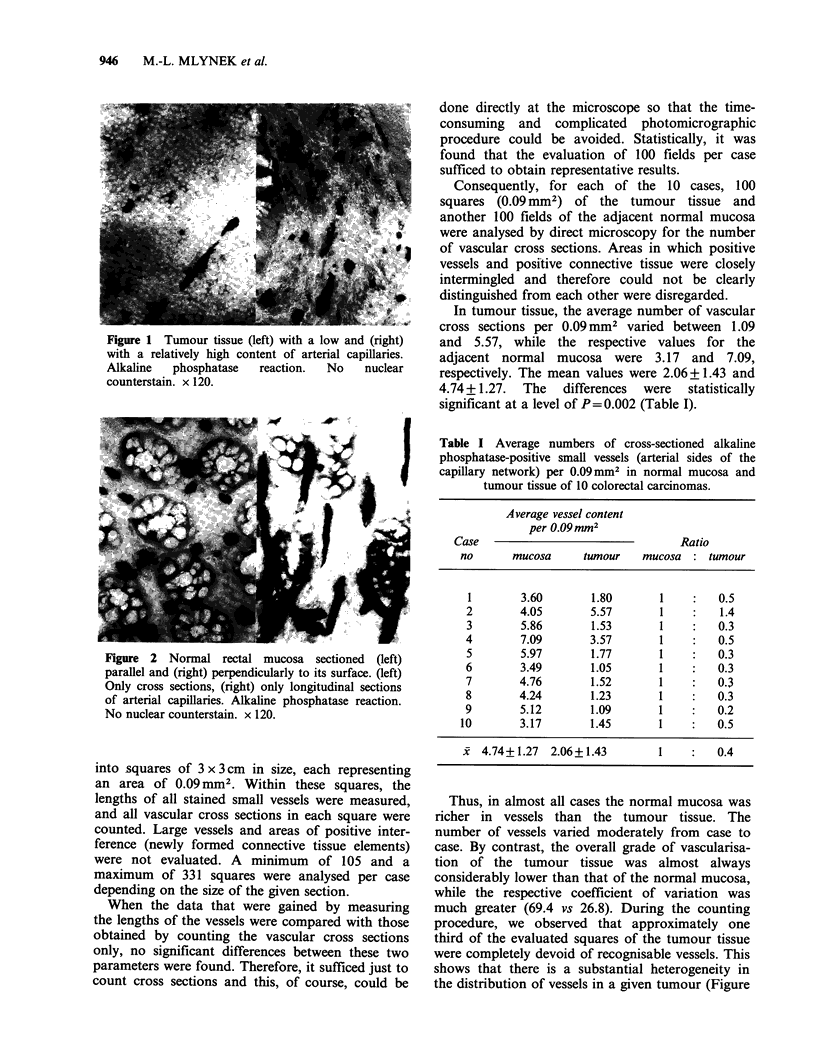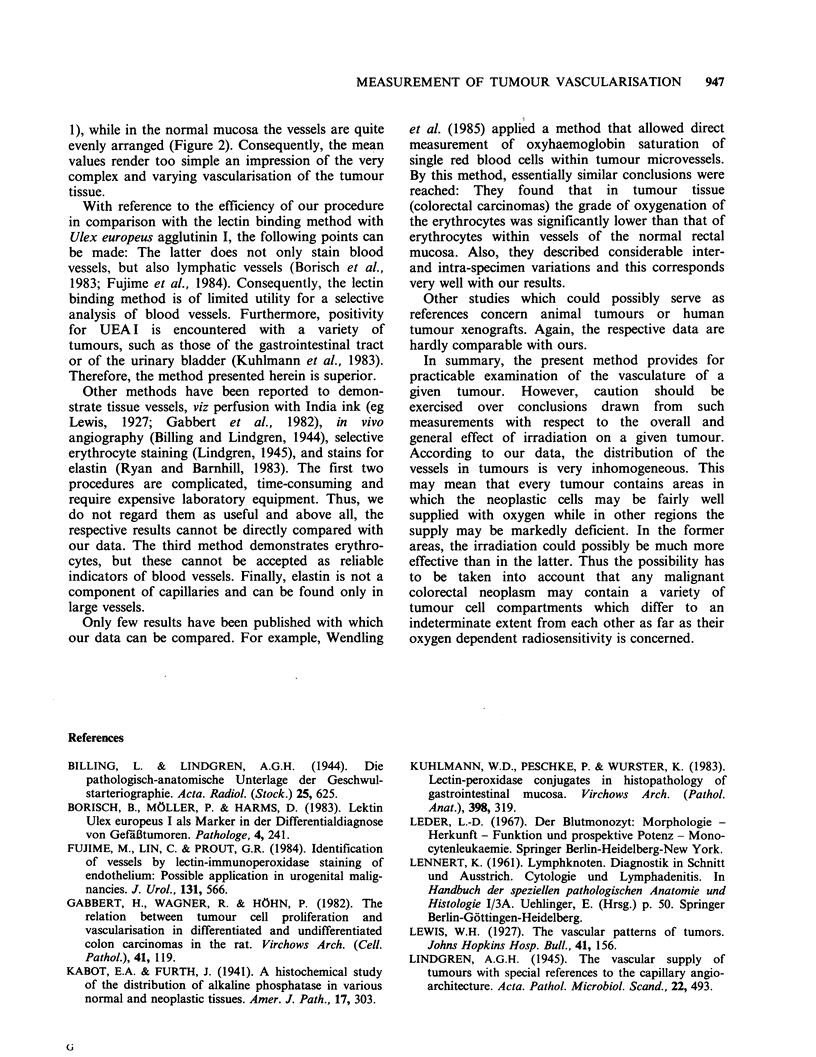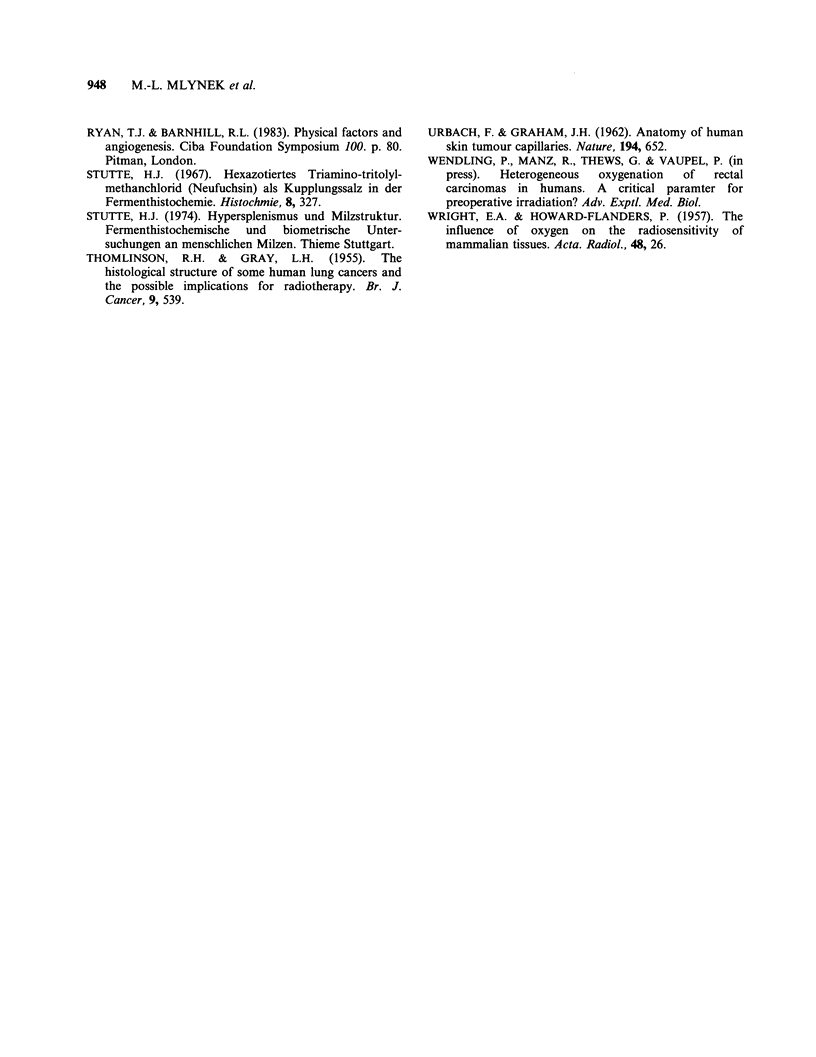# Measurement of the grade of vascularisation in histological tumour tissue sections.

**DOI:** 10.1038/bjc.1985.282

**Published:** 1985-12

**Authors:** M. L. Mlynek, D. van Beunigen, L. D. Leder, C. Streffer

## Abstract

**Images:**


*Br. J. Cancer* (1985), **52**, 945–948

**Short Communication**

### Measurement of the grade of vascularisation in histological tumour tissue sections

M.-L. Mlynek[1], D. van Beunigen[2], L.-D. Leder[1] & C. Streffer[2]

[1]*Institute of Pathology, University of Essen, D-4300 Essen;* [2]*Institute of Medical Radiation Physics and Radiobiology, Department of Medical Radiobiology, University of Essen, D-4300 Essen, FRG*

The grade of vascularisation of malignant tumours as well as their oxygen supply are factors of vital interest, since the response of tumour cells to irradiation may be modified and influenced by them (eg Thomlinson & Gray, 1955; Wright & Howard-Flanders, 1957). However, precise data on the vascularity of human neoplastic tissues are largely lacking in the literature, probably because there is no reliable and readily available method for its measurement.

We have therefore developed a procedure with which to obtain fairly substantial data on the number of vessels in a given tissue wherein use is made of a histochemical procedure for the demonstration of alkaline phosphatase, an enzyme present quite distinctively in the endothelial cells of the arterial part of the terminal vascular system (Kabot & Furth, 1941; Stutte, 1974; Urbach & Graham, 1962; Lennert, 1961; Leder, 1967).

Fresh frozen sections were cut at 5 $\mu$m from unfixed tissue of 10 cases of colorectal carcinoma. Depending on the size of a given tumour, 2–6 tissue blocks were prepared in each of the cases. For comparison and control, tissue from the adjacent normal mucosa was also sectioned.

The sections were incubated for 30 min in the following medium (Stutte, 1967): 4 drops (0.2 ml) of a 5% solution of sodium nitrite in distilled water and 4 drops (0.2 ml) of a 5% solution of triamino-tritolyl-methanechloride (Fuchsin B) in 2N HCl were mixed and diazotised for 1 min. Then, 40 ml of 0.05 M propanediol buffer, pH 9.75, were added. The pH was adjusted to 9.4 with 2N NaOH, and 10 mg of naphthol AS-BI phosphate, dissolved in 1 ml of dimethylformamide, were admixed. The mixture was filtered into a coplin jar. After incubation, the slides were thoroughly rinsed in tap water and mounted in glycerine jelly.

Positive structures were stained brilliant red with varying intensity depending on their enzymatic activity. The reaction product was amorphous and revealed a precise localisation. There was a yellow background staining but no formation of interfering cyrstals.

In tumour tissue, the small arterioles as well as the arterial parts of the capillaries stood out very clearly and were easily discernible and identifiable because they exhibited the strongest positivity of all the tissue elements. The distribution of the vessels was relatively inhomogeneous and irregular (Figure 1). In some areas, not only were the vessels positive, but also parts of the newly formed stromal connective tissue. In such areas it was sometimes impossible to identify positive vessels clearly and to distinguish them from positive connective tissue elements. Some neutrophils were also reactive but much weaker than the vessels. Occasionally, a few large vessels with wide lumina were observed, and in rare cases a few tumour cells were weakly positive. However, these components did not interfere with positive vessels because of the extremely strong reaction of the latter and because of the profound morphological differences between positive capillaries and other positive elements.

In contrast to the tumour tissue, the normal mucosa exhibited a very regular vascular pattern. If the mucosa was cut parallel to its surface, only vascular cross sections could be detected, while sections that were cut perpendicularly to the mucosal surface disclosed only longitudinal vascular sections (Figure 2). Usually, however, most parts of the normal mucosa were cut transversely resulting in a mixture of cross sections and longitudinal sections.

Although alkaline phosphatase is stained only on the arterial side of the capillary network, the method can be accepted as providing data representative of the total vascularisation, because any functionally active capillary network must contain both an arterial and a venous side, otherwise there would be no blood flow.

For pre-evaluation, the entire area of one section per case was photographed at a magnification of ×98. All photomicrographs were put together, which resulted in a sort of map of the respective tissue section. This panoramic picture was divided

Correspondence: M-L. Mlynek
Received 3 April 1985; and in revised form, 15 July 1985.

© The Macmillan Press Ltd., 1985

**Figure 1** Tumour tissue (left) with a low and (right) with a relatively high content of arterial capillaries. Alkaline phosphatase reaction. No nuclear counterstain. × 120.

**Figure 2** Normal rectal mucosa sectioned (left) parallel and (right) perpendicularly to its surface. (left) Only cross sections, (right) only longitudinal sections of arterial capillaries. Alkaline phosphatase reaction. No nuclear counterstain. × 120.

into squares of 3 × 3 cm in size, each representing an area of 0.09 $mm^2$. Within these squares, the lengths of all stained small vessels were measured, and all vascular cross sections in each square were counted. Large vessels and areas of positive interference (newly formed connective tissue elements) were not evaluated. A minimum of 105 and a maximum of 331 squares were analysed per case depending on the size of the given section.

When the data that were gained by measuring the lengths of the vessels were compared with those obtained by counting the vascular cross sections only, no significant differences between these two parameters were found. Therefore, it sufficed just to count cross sections and this, of course, could be done directly at the microscope so that the time-consuming and complicated photomicrographic procedure could be avoided. Statistically, it was found that the evaluation of 100 fields per case sufficed to obtain representative results.

Consequently, for each of the 10 cases, 100 squares (0.09 $mm^2$) of the tumour tissue and another 100 fields of the adjacent normal mucosa were analysed by direct microscopy for the number of vascular cross sections. Areas in which positive vessels and positive connective tissue were closely intermingled and therefore could not be clearly distinguished from each other were disregarded.

In tumour tissue, the average number of vascular cross sections per 0.09 $mm^2$ varied between 1.09 and 5.57, while the respective values for the adjacent normal mucosa were 3.17 and 7.09, respectively. The mean values were $2.06 \pm 1.43$ and $4.74 \pm 1.27$. The differences were statistically significant at a level of $P = 0.002$ (Table I).

**Table I** Average numbers of cross-sectioned alkaline phosphatase-positive small vessels (arterial sides of the capillary network) per 0.09 $mm^2$ in normal mucosa and tumour tissue of 10 colorectal carcinomas.

| *Case no* | *Average vessel content per 0.09 mm²* *mucosa* | *tumour* | *Ratio* *mucosa* | : | *tumour* |
|---|---|---|---|---|---|
| 1 | 3.60 | 1.80 | 1 | : | 0.5 |
| 2 | 4.05 | 5.57 | 1 | : | 1.4 |
| 3 | 5.86 | 1.53 | 1 | : | 0.3 |
| 4 | 7.09 | 3.57 | 1 | : | 0.5 |
| 5 | 5.97 | 1.77 | 1 | : | 0.3 |
| 6 | 3.49 | 1.05 | 1 | : | 0.3 |
| 7 | 4.76 | 1.52 | 1 | : | 0.3 |
| 8 | 4.24 | 1.23 | 1 | : | 0.3 |
| 9 | 5.12 | 1.09 | 1 | : | 0.2 |
| 10 | 3.17 | 1.45 | 1 | : | 0.5 |
| $\bar{x}$ | $4.74 \pm 1.27$ | $2.06 \pm 1.43$ | 1 | : | 0.4 |

Thus, in almost all cases the normal mucosa was richer in vessels than the tumour tissue. The number of vessels varied moderately from case to case. By contrast, the overall grade of vascularisation of the tumour tissue was almost always considerably lower than that of the normal mucosa, while the respective coefficient of variation was much greater (69.4 *vs* 26.8). During the counting procedure, we observed that approximately one third of the evaluated squares of the tumour tissue were completely devoid of recognisable vessels. This shows that there is a substantial heterogeneity in the distribution of vessels in a given tumour (Figure

1), while in the normal mucosa the vessels are quite evenly arranged (Figure 2). Consequently, the mean values render too simple an impression of the very complex and varying vascularisation of the tumour tissue.

With reference to the efficiency of our procedure in comparison with the lectin binding method with *Ulex europeus* agglutinin I, the following points can be made: The latter does not only stain blood vessels, but also lymphatic vessels (Borisch *et al.*, 1983; Fujime *et al.*, 1984). Consequently, the lectin binding method is of limited utility for a selective analysis of blood vessels. Furthermore, positivity for UEA I is encountered with a variety of tumours, such as those of the gastrointestinal tract or of the urinary bladder (Kuhlmann *et al.*, 1983). Therefore, the method presented herein is superior.

Other methods have been reported to demonstrate tissue vessels, *viz* perfusion with India ink (eg Lewis, 1927; Gabbert *et al.*, 1982), *in vivo* angiography (Billing and Lindgren, 1944), selective erythrocyte staining (Lindgren, 1945), and stains for elastin (Ryan and Barnhill, 1983). The first two procedures are complicated, time-consuming and require expensive laboratory equipment. Thus, we do not regard them as useful and above all, the respective results cannot be directly compared with our data. The third method demonstrates erythrocytes, but these cannot be accepted as reliable indicators of blood vessels. Finally, elastin is not a component of capillaries and can be found only in large vessels.

Only few results have been published with which our data can be compared. For example, Wendling *et al.* (1985) applied a method that allowed direct measurement of oxyhaemoglobin saturation of single red blood cells within tumour microvessels. By this method, essentially similar conclusions were reached: They found that in tumour tissue (colorectal carcinomas) the grade of oxygenation of the erythrocytes was significantly lower than that of erythrocytes within vessels of the normal rectal mucosa. Also, they described considerable inter- and intra-specimen variations and this corresponds very well with our results.

Other studies which could possibly serve as references concern animal tumours or human tumour xenografts. Again, the respective data are hardly comparable with ours.

In summary, the present method provides for practicable examination of the vasculature of a given tumour. However, caution should be exercised over conclusions drawn from such measurements with respect to the overall and general effect of irradiation on a given tumour. According to our data, the distribution of the vessels in tumours is very inhomogeneous. This may mean that every tumour contains areas in which the neoplastic cells may be fairly well supplied with oxygen while in other regions the supply may be markedly deficient. In the former areas, the irradiation could possibly be much more effective than in the latter. Thus the possibility has to be taken into account that any malignant colorectal neoplasm may contain a variety of tumour cell compartments which differ to an indeterminate extent from each other as far as their oxygen dependent radiosensitivity is concerned.

### References

BILLING, L. & LINDGREN, A.G.H. (1944). Die pathologisch-anatomische Unterlage der Geschwulstarteriographie. *Acta. Radiol.* (*Stock.*) **25**, 625.

BORISCH, B., MÖLLER, P. & HARMS, D. (1983). Lektin Ulex europeus I als Marker in der Differentialdiagnose von Gefäßtumoren. *Pathologe*, **4**, 241.

FUJIME, M., LIN, C. & PROUT, G.R. (1984). Identification of vessels by lectin-immunoperoxidase staining of endothelium: Possible application in urogenital malignancies. *J. Urol.*, **131**, 566.

GABBERT, H., WAGNER, R. & HÖHN, P. (1982). The relation between tumour cell proliferation and vascularisation in differentiated and undifferentiated colon carcinomas in the rat. *Virchows Arch.* (*Cell. Pathol.*), **41**, 119.

KABOT, E.A. & FURTH, J. (1941). A histochemical study of the distribution of alkaline phosphatase in various normal and neoplastic tissues. *Amer. J. Path.*, **17**, 303.

KUHLMANN, W.D., PESCHKE, P. & WURSTER, K. (1983). Lectin-peroxidase conjugates in histopathology of gastrointestinal mucosa. *Virchows Arch.* (*Pathol. Anat.*), **398**, 319.

LEDER, L.-D. (1967). Der Blutmonozyt: Morphologie – Herkunft – Funktion und prospektive Potenz – Monocytenleukaemie. Springer Berlin-Heidelberg-New York.

LENNERT, K. (1961). Lymphknoten. Diagnostik in Schnitt und Ausstrich. Cytologie und Lymphadenitis. In *Handbuch der speziellen pathologischen Anatomie und Histologie* I/3A. Uehlinger, E. (Hrsg.) p. 50. Springer Berlin-Göttingen-Heidelberg.

LEWIS, W.H. (1927). The vascular patterns of tumors. *Johns Hopkins Hosp. Bull.*, **41**, 156.

LINDGREN, A.G.H. (1945). The vascular supply of tumours with special references to the capillary angioarchitecture. *Acta. Pathol. Microbiol. Scand.*, **22**, 493.

RYAN, T.J. & BARNHILL, R.L. (1983). Physical factors and angiogenesis. Ciba Foundation Symposium *100*. p. 80. Pitman, London.

STUTTE, H.J. (1967). Hexazotiertes Triamino-tritolylmethanchlorid (Neufuchsin) als Kupplungssalz in der Fermenthistochemie. *Histochmie*, **8**, 327.

STUTTE, H.J. (1974). Hypersplenismus und Milzstruktur. Fermenthistochemische und biometrische Untersuchungen an menschlichen Milzen. Thieme Stuttgart.

THOMLINSON, R.H. & GRAY, L.H. (1955). The histological structure of some human lung cancers and the possible implications for radiotherapy. *Br. J. Cancer*, **9**, 539.

URBACH, F. & GRAHAM, J.H. (1962). Anatomy of human skin tumour capillaries. *Nature*, **194**, 652.

WENDLING, P., MANZ, R., THEWS, G. & VAUPEL, P. (in press). Heterogeneous oxygenation of rectal carcinomas in humans. A critical paramter for preoperative irradiation? *Adv. Exptl. Med. Biol.*

WRIGHT, E.A. & HOWARD-FLANDERS, P. (1957). The influence of oxygen on the radiosensitivity of mammalian tissues. *Acta. Radiol.*, **48**, 26.